# Her Heart's Mistake

**Published:** 1889-04-20

**Authors:** E. D. Cumming

**Affiliations:** Author of "An Ordeal by Silence"


### Her Heart's Mistake.

By E. D. Cumming, Author of "An Ordeal by Silence."

*(Continued from page 31.)*

Chapter II.—*(Continued.)*

The solemn-faced khitmugars stood behind their master's chairs, or bustled about for dishes and plates as earnestly as though they were waiting at a state dinner party, marvelling the while at the inscrutable ways of a people who would deliberately plan and carry out so mad a freak as this. What pleasure could there be in riding all the way out here at night to have an uproarious feast and ride back again? It was very unaccountable the pleasure they derived from the proceeding; the continuous lively buzz of conversation was only interrupted by the clattering of knives and forks, and bursts of laughter which made the walls ring. Everyone was good humoured and gay. Captain Bairdsley had found a place beside the hostess, and was exerting himself to entertain her. The love-lorn Tops, with infinite craftiness, had secured a seat next to Kate, and was dividing his attention between cold teal and her face. "Particular friends" had taken care to sit together, and the unattached men seem quite satisfied with their lot. The night wore on apace, and when supper was over cheroots were lighted by request "to keep away the mosquitoes"; and the call to Colonel Tenterdale for a song was responded to with great goodwill, if little musical talent. Then Mr. Allen, of the Public Works Department, was desired to give a song with a chorus; and, in his turn, Captain Bairdsley sang "Killarney," which was rapturously encored. It was past midnight when Mr. Strachan got up and proposed the health of Mr. and Mrs. Pringle by way of bringing "the charming picnic they have given us" to an end. And then horses and carriages were brought round, the servants were left to pack up glass and crockery to go back in the bullock carts, and the merry crowd set out for the homeward journey. Mr. Wentworth started with Miss Harrison, and waxed eloquent on the delights of a fast gallop by moonlight; for his jealous eye had detected Captain Bairdsley and Mrs. Tenterdale in the immediate rear. But Kate was not in a mood to hurry, and before they had gone two miles, Mrs. Tenterdale and her cavalier came up and joined them. By what exact process of feminine diplomacy it was accomplished, Tops could not have explained; but ten minutes later he was careering along in attendance upon the elder lady, leaving the other two far behind.

"Are you tired, Miss Harrison?" asked Bairdsley. "Would you prefer to ride on with Mrs. Tenterdale?"

"No, thank you," said Kate; "I'm not tired, but I think I will jog home quietly. Pray don't let me keep you back if you want to go with the others."

There was no one to hear his answer except the horses and a brown owl, which was flitting among the trees, but Bairdsley lowered his voice to reply; and they rode home to cantonments together.

"Good-night," he said, taking her hand again when he had lifted her from her horse.

"Good-night," she replied, softly; and she looked up in his eyes as she spoke, with a glance that made every nerve in his frame tingle.

He left the horse at the doctor's stable, and walked home to his bungalow. The pariahs, baying in lusty chorus in the distant bazaar, and the jackals' weird discordant screams did their best to make night hideous. But he scarcely heard them; his ears were still repeating Kate's "Good-night."

Chapter III. "People are Talking."

"Yes," said Mrs. Tenterdale, who was the central figure of a little group of ladies on the club verandah, "I am very fond of Kate Harrison, and take a great interest in her. I should like nothing better than to see her happily married."

"Don't you think that eighteen is rather too young for a girl to marry?" asked Mrs. Pringle.

"It depends entirely on the girl herself. Kate is old for her age, and has a vast amount of common sense."

"We must remember that she has only been out here a year, and was buried alive in the country at home before. No one has a higher opinion of Kate's good sense than I have; but I cannot think she has seen enough of the world to know her own mind thoroughly," said Mrs. Pringle.

"I don't think she could hope to find a more desirable party than Captain Bairdsley," said Mrs. Arkwright, "and he has certainly paid her unmistakably marked attention. It's a long time since we had a wedding in Meerut. I like weddings."

"It looks as though Captain Bairdsley was inclined to gratify your taste," laughed Mrs. Tenterdale, "and for my part I hope he will."

The Colonel's wife was one of those well-meaning, kindly, but rather intrusive people found everywhere, who had a keen eye for what she was wont to describe as "a case." If any young man of marriageable age betrayed a liking for any particular young lady's society, Mrs. Tenterdale at once conceived a warm interest in and sympathy for him, and did her utmost to throw the two together. She would ask them to come to chota haziri (the early morning meal of India) and go for a ride with her afterwards. And when Mr. Jones and Miss Brown arrived at her bungalow they were certain to find one other man there, whose special mission was to relieve them of Mrs. Tenterdale's too direct chaperonage by riding with her. They would be asked to afternoon tea together, or invited to dinner, and carefully placed side by side. Miss Brown would be asked to drive out with her match-making friend in the evening, and it was passing strange if Mr. Jones was not picked up at the polo-ground or elsewhere before they returned home. Since the day when Captain Bairdsley paid such an unconscionably long visit at the corner bungalow she had watched him carefully, and her easily-awakened suspicions were soon confirmed; and if the gentleman was not allowed to have Kate entirely to himself at the band on the tennis-court, it was not Mrs. Tenterdale's fault. An intruder of either sex might join the pair, and be allowed to remain with them for ten minutes undisturbed; but if the Colonel's wife happened to be near, the trio then became a quartette, and in good time the quartette dissolved itself into two duets through the good lady's judicious manœuvreing.

"Captain Bairdsley seems to be a good deal at your house, Mrs. Brent," she remarked one evening as they sat in Dr. Harrison's carriage, driving slowly up and down the Mall.

"Yes, Mrs. Tenterdale, my niece and he are very fond of singing together; their voices blend exceedingly well."

"He appears to admire Kate very much."

"Do you think so? I had not noticed it, but I can't say I am very observant about such matters."

"Why, my dear Mrs. Brent, it must have been obvious to the whole station how frequently they are together. I hear people talking about it, and coupling their names without the least reserve, every day."

"I did not know it," said Mrs. Brent, who was not a very far-seeing woman. "Kate often speaks about Captain Bairdsley, but she has never said anything which would have led me to imagine that she cared for him."

"Well now, you are all coming to dine with us to-night, and the Captain will be there, too. I won't let him sit next to Kate at dinner, and you will see if he does not go straight to her side as soon as the men come in from the dining-room afterwards."

So Mrs. Brent, who would have been quite contented to let matters take their own way, was goaded into watching Captain Bairdsley's behaviour for that evening at least, and what she saw, added to what she had heard from Mrs. Tenterdale, prompted her to speak to her niece on the subject. Hadn't the Captain been paying her a good deal of attention? she asked, very straightforwardly. Miss Harrison didn't know that he had; but he was always very nice and kind to her. Well, it had come to her aunt's knowledge that every one in Meerut was talking about them, and really, unless there was something it, Kate ought to be careful. It didn't do for a girl's name to be in everyone's mouth like that. Kate was perfectly certain she had done nothing she need be ashamed of, she frankly confessed that she liked Captain Bairdsley and thought that he liked her. But she would remember what Aunt Agnes had said, and not give any cause for idle gossip.

But as Miss Harrison lay on her cane lounge under the punkah that day, she caught herself reading the same page of "Robert Elsmere" over and over again, without the least idea of what the writer said. George Bairdsley's face came before her eyes; George Bairdsley's voice murmured in her ear, and now and then her aunt's words, "everyone in

Meerut is talking about you," rose to her memory. She dropped her book and closed her eyes to think. Yes; he had been very much with her lately; a great deal more than any man had ever been before. She recalled that long moonlight ride, when he had been content to stay at her side without exchanging a word; and his "Good-night, Kate," when they parted. Yes, since that night there had been much in his manner to make idle people talk. At the band, he was sure to be among the first to come up to the carriage. When other men were playing pool in the club, or smoking outside, he always found his way across the compound to the ladies' rooms before she had been there very long. And that night when they dined with the Tenterdale's, and he had been seated far away down the other side of the table, she had never once looked up without catching his eye; beyond all doubt she was something more to him than a friend. And how did she regard him? she asked herself. Supposing that he were ordered away to a distant station to-morrow, how would she regard the prospect of losing him? Could she bid him a cheery good-bye and let him pass out of her mind just as he passed out of sight? No; she could not, she answered. She had never felt towards anyone as she did towards this man. The little informal dances the 110th gave every week would be nothing to her if he were not there. Those new songs from Harold's, the Calcutta musicsellers, would be empty sound if he were not standing by the piano while she sang. He was so much older than she was; had seen so much more of the world than she. Mr. Wentworth had told her how he was already an authority on games and sports at the mess, though he had been with them barely a month. At billiards, at polo, at the pigeon-shooting ground, he held his own against all. He was a man amongst men. She was proud to be seen with him, proud that her name should be connected with his. She loved him, and he loved her, she was sure. Meerut might chatter and gossip as it pleased; she would do nothing that would give anyone excuse for saying that she overstepped the bounds of propriety by a hair's-breadth, but she would not raise a finger or speak a word to drive George Bairdsley from her. Why should she? She was not ashamed of loving him, or of his admiration for her. Did the old women expect her to play at repelling his advances; to coyly avoid him, and lead him on to pursue the prize he knew could be his when he asked for it? No; they might think her bold and unwomanly if they pleased, but George Bairdsley and she had looked into each other's eyes too often now to act such a farce as that. And then she opened her book once more, thinking to dismiss the subject from her mind; but it would not do. She lost the thread of the story before she had perused ten lines, and resigned herself to her thoughts again. It was George Bairdsley—always George Bairdsley. No book ever written was sufficiently interesting to oust his image, and she smiled to herself as she thought that at this moment he was in the little bungalow over yonder, day-dreaming of her.

Dr. Harrison, busy all day and every day at the hospital, had heard little or nothing of the talk to which Captain Bairdsley's frequent visits to his house had given rise. But one morning, when Kate was absent on her morning ride with that gentleman, Mrs. Brent felt it her duty to enlighten him as to what Mrs. Tenterdale and others were saying. The Doctor passed the matter lightly by; he knew how little was required to furnish food for gossip in India. Kate was a pretty girl, and might expect to be made a mark for people's tongues if a man ventured to look at her. Nevertheless, he quietly studied his daughter's demeanour for a day or two, and came to the conclusion that he could detect some little change in her bearing. She was cheerful and happy, but sometimes when she was working with her needle or reading she would let her hands fall, and relapse into a state of dreamy thought which he had never observed before. She had fits of abstraction at meals, and started when spoken to, as though awakened from sleep. These signs seemed to portend something, and the Doctor was roused to action. She was his only child, the sunshine of his life since her mother died, and he must know what was on her mind. He took advantage of an occasion, when his sister was confined to her room with a touch of fever, to sound Kate, cautiously at first, about this admirer of hers. It was not in her nature to parry enquiries from such a source, and she admitted that she did like Captain Bairdsley; she liked him better than anyone else, and thought that he liked her. Of the truth of the latter opinion her father had no doubt whatever; it was the most natural thing in the world, to his mind, that any man who knew Kate should fall in love with her. It was only regarding her feelings that he wished to inform himself, and since she appeared to like the man, he must make sure that he was worthy of her before the affair was allowed to go too far.

The Doctor had passed the greater part of his service in India, but he had never liked the country. His ambition had always been to retire early, and settle down to spend the evening of his days at Cheltenham or Bath, with Kate near him. He longed for the time to come when he might shake off harness and leave the country for ever, for he knew that men who passed their lives there found England, when they returned, a land more strange than that in which their life's work had lain. He had had many friends at home at one time, but as the years rolled by they dropped out of the ranks one by one, and the Doctor felt that with each death another link with the old country was broken.

He had held remunerative appointments, and had saved a few thousand pounds, which he had invested profitably with his long-cherished scheme in view. He must return before he grew too old to renew the few remaining friendships, before he lost touch altogether with his native land, and Kate must come with him, or at least follow him soon. He had always dreaded the risk of her becoming attached to a civilian or an officer in the Indian Army, marriage to whom would imply fixed residence in the East. Her eyes were turned towards home almost as longingly as her father's, though she entered now with all the zest of youth and health into the delights of an Indian station. But that could not always be. The time must come when these pleasures would lose their charms, and with them would disappear all the attractions the country held for her. She must marry a man who could bring her home ere she became early old and withered.

He ought to learn all he could, then, about this Captain Bairdsley at once; if he possessed means of his own, enough to enable him to support a wife, it would be all right. But possibly he had nothing but his pay, and meant to join the Indian Staff Corps, whose higher emoluments would place him in a position to marry, and that would be an absolute disqualification, an absolute bar to his union with Kate.

"But," reflected the doctor, puffing a cloud of smoke towards the ceiling, "I'm going a bit too fast. *I* don't know that the man wants to marry her. He may be amusing himself for aught I can tell, and will transfer his attentions to some other girl when the hill stations send down their crowds for the winter. I'll see Tenterdale, and ask him privately if he can give me any information about Bairdsley. He has only just joined the regiment, but the Colonel may know something about his means and antecedents; and there seems to be good ground to justify my questioning him on the subject."

Although Dr. Harrison reassured himself thus, the idea of allowing even so intimate a friend as Colonel Tenterdale to suppose that he was in haste to believe that Captain Bairdsley was wooing Kate, was distasteful to him; and though he met the Colonel regularly every evening at the club or elsewhere, several days slipped by before anything passed between them on the matter. But it chanced one evening that, as they stood talking together at the band, Kate rode into the gardens accompanied by her admirer, and the Colonel noticed their approach.

"It appears to me, Harrison," he remarked, half seriously, "that the old women are right." He nodded in the direction of the young people as he spoke, and Dr. Harrison saw that his opportunity had come.

"Boys and girls will play, Colonel; we can't stop them," he said, lightly.

"To be honest with you, Doctor, I don't believe it is play—on Bairdsley's side, at all events. What reason have you to think that he is in earnest?

"Something Phelps told my wife the other day. It seems that he was chaffing Bairdsley about his devotion to your daughter in their bungalow one day, as he had done once or twice before, and Bairdsley suddenly turned on him with a face like a turkey cock and begged him to drop the subject."

"And Phelps told your wife this?"

"Yes; he's her cousin you know," added the Colonel, by way of extenuating Captain Phelps' want of reticence.

"Do you know anything about Bairdsley, Colonel? I mean regarding his personal character and antecedents?" enquired Dr. Harrison, after a pause.

"Very little; you see he only joined us a month ago from the other battalion, and none of our fellows knew him. I

don't know who or what his people are, but he has more money than most of us, and isn't backward about spending it."

"Indeed; I wonder what induced a man of means to come out to this country when he could have stayed at home."

"He seems to be a keen soldier; he's the smartest officer I have, anyway, and I imagine that the rumours of probable war on the frontier brought him out, as they have many others who have a fancy for the smell of powder."

"You say he appears to have money?" said Dr. Harrison, harking back to the Colonel's previous remark. "After what you have told me about his being in earnest, I don't make any apology for my curiosity."

"I quite recognise your feeling in the matter, Doctor, and will gladly tell you all I know, but it isn't much. Bairdsley certainly has means of his own; he gave a hundred and fifty rupees last week to a subscription we are getting up for Sergeant Williams's widow—the poor fellow who blew his brains out in a paroxysm of fever the other day—and he is always ready to open his purse for our regimental charities. I hear he is paying two thousand for a couple of walers someone at Saharanpore wants to sell. A man can't do things of that kind without money at his back."

"No," said the Doctor; "Australian horses at a thousand apiece are not luxuries in which the British soldier can generally indulge. What kind of man is he personally? I hardly know him myself; he has dined at my house three or four times, but there have always been other men there, and I have never talked to him by myself."

"He's a very pleasant companion. All the other fellows swear by him. First-rate horseman, and a thundering good shot. Not domestic accomplishments, perhaps, but it shows there must be something in him. Ah, there's my wife signalling to me, and I must be off! Good night, Doctor!" And Colonel Tenterdale strode away towards his dogcart, in which his wife was seated awaiting him.

Dr. Harrison did not go over to the club for his customary rubber of whist when his friend left him. He had heard enough to give him food for serious thought; this man Bairdsley seemed to be "in earnest" in his attentions to Kate, and was evidently in a position to marry her if he wished. And though the Doctor told himself half-a-dozen times in the course of the lonely walk he took that evening that he was exercised in mind without due reason—that he was building a house of cards, and was no wiser after all than the station gossips, who professed to believe that his daughter's engagement was an unannounced fact—he could not but feel some anxiety. It was a very possible contingency beyond all doubt, and one upon which the future happiness of his daughter might depend. She was very young—only a little over eighteen, and he had hardly expected to part with her just yet. Well, well, it was really too soon to take it for granted that he would be asked to do so. Time would show whether the gossips were right or not; and as the Doctor thus consoled himself he turned into his own compound, and saw his sister and Kate on the verandah.

"You didn't come to the club this evening, papa," said the latter as he joined them.

"No, dear; I went for a stroll instead."

Kate looked at her father anxiously; he was one of those men, the honour of the medical profession, who never spared himself, and this evening he looked distraught and tired.

"You're working too hard, daddy. Can't you give yourself a little rest, now that Dr. Anson has come back? Couldn't you take a few weeks' leave to the hills?"

"I'm all right, my dear, and there is quite enough work for us all just now, with nearly every bed in the wards occupied. But wouldn't you like to take a run up to Mussoorie for a month with your aunt?" The Doctor looked at her narrowly as he asked the question; her answer would give a better clue to her feeling for Captain Bairdsley than the acknowledgment that she "liked him," which might mean much or nothing.

"I wouldn't care to leave you, daddy," she said with a slight tremor in her voice. "At least not just now." The verandah was in darkness save for the light of one dim lamp at the far end, but the Doctor saw the colour rise to her cheeks as she spoke, and knew its import.

"Well, I know what to expect," said he to himself as he brushed aside the purdah and went into his own room. "I'll try and improve my acquaintance with Bairdsley this evening if I can get a chance."

It was the 100th's "guest night," and the Doctor was to dine at their mess. He did not go out very often, for after the day's work he was glad to spend the after-dinner hours in his chair, listening to Kate's music or reading a book, and late nights are not the rule in a country where the working day begins at 6 a.m.

*(To be continued.)*

